# Potassium Ion Channels in Glioma: From Basic Knowledge into Therapeutic Applications

**DOI:** 10.3390/membranes13040434

**Published:** 2023-04-15

**Authors:** Samar Younes, Nisreen Mourad, Mohamed Salla, Mohamad Rahal, Dalal Hammoudi Halat

**Affiliations:** 1 Department of Biomedical Sciences, School of Pharmacy, Lebanese International University, Bekaa 146404, Lebanon; 2 Institut National de Santé Publique, d’Épidémiologie Clinique et de Toxicologie-Liban (INSPECT-LB), Beirut 1103, Lebanon; nisreen.mourad@liu.edu.lb; 3 Department of Pharmaceutical Sciences, School of Pharmacy, Lebanese International University, Bekaa 146404, Lebanon; mohamad.rahal@liu.edu.lb (M.R.);; 4 Department of Biological and Chemical Sciences, School of Arts and Sciences, Lebanese International University, Bekaa 146404, Lebanon; mohammad.salla@liu.edu.lb; 5 Academic Quality Department, QU Health, Qatar University, Doha 2713, Qatar; dalal.hammoudi@liu.edu.lb

**Keywords:** potassium channels, glioma, proliferation, apoptosis, migration, potassium channel inhibitors, drug repurposing

## Abstract

Ion channels, specifically those controlling the flux of potassium across cell membranes, have recently been shown to exhibit an important role in the pathophysiology of glioma, the most common primary central nervous system tumor with a poor prognosis. Potassium channels are grouped into four subfamilies differing by their domain structure, gating mechanisms, and functions. Pertinent literature indicates the vital functions of potassium channels in many aspects of glioma carcinogenesis, including proliferation, migration, and apoptosis. The dysfunction of potassium channels can result in pro-proliferative signals that are highly related to calcium signaling as well. Moreover, this dysfunction can feed into migration and metastasis, most likely by increasing the osmotic pressure of cells allowing the cells to initiate the “escape” and “invasion” of capillaries. Reducing the expression or channel blockage has shown efficacy in reducing the proliferation and infiltration of glioma cells as well as inducing apoptosis, priming several approaches to target potassium channels in gliomas pharmacologically. This review summarizes the current knowledge on potassium channels, their contribution to oncogenic transformations in glioma, and the existing perspectives on utilizing them as potential targets for therapy.

## 1. Introduction to Ion Channels

In various aspects of life, the bioelectrical signals arising from the activity of ion channels are fundamental for different cellular processes and functions. Ion channels are transmembrane proteins that create a regulated pore structure through which ions can pass across the lipid bilayer of biological membranes [1,2]. When such an aqueous pore is open, ions move freely between cellular compartments, and this ion fluctuation can impact an armamentarium of pathways, including electrical excitation, signal transduction, regulation of secretion, and contractility, in addition to mechanisms that preserve normal tissue homeostases, such as cell proliferation, differentiation, migration, and apoptosis [3,4].

Despite spanning a different array of families, ion channels may be broadly categorized into voltage-gated channels and ligand-gated channels based on activation mechanisms and structural similarities, and such attributes and properties have been extensively reviewed [5,6,7]. With the transportation of ions having a critical role in cellular physiology, it is well known that the malfunction of ion channels can fundamentally lead to many diseases. In addition to signal transmission in nerves and muscle contraction, ion channels regulate brain activity, insulin secretion, water and ion transport, immune function, and others. Being ubiquitous and selective, they often require precise stereochemistry, with even subtle changes to their structures resulting in unfavorable physiological consequences [8]. With the wide distribution of channels and their remarkable roles, as well as the disorders associated with their structural or physiological malfunction, they have historically served as potential drug targets [9]. Not only have ion channel-targeting drugs been exploited in neuronal and cardiac diseases, but other nonclassical therapeutic benefits have emerged, including cystic fibrosis, smoking cessation, diabetes, and cancer [10]. Interestingly, lysosomal ion channels [11], mitochondrial ion channels [12], and Piezo channels [13] are also being prominently studied for their role in health and disease and represent a yet unraveled potential for a new era of therapeutics. In what follows, this review focuses on the importance of ion channels in cancer, with the role of potassium channels of the cell membrane in glioma approached in detail.

## 2. The Role of Ion Channels in Cancer

Voltage-sensitive ion channels have become a focus of research on cancer development into a more malignant phenotype. In cancer, various channels were found to be expressed in different cancer types, whereby they play major roles in cell proliferation, migration, invasion, and survival, as evidenced by the increased expression or increased kinetics of these channels upon malignant transformation [14]. As a result, these channels now represent promising directions for cancer therapy, whereby the blockade or reduction in their activity may be a strategy to prevent or treat oncological disorders [15]. Besides regulating several features of cancer cell behavior, ion channels are considered to be novel cancer biomarkers and show the potential to be exploited for diagnostic, prognostic, or predictive purposes [16]. With their location within the plasma membrane and the multiple layers of regulation they possess, ion channels represent key clinical targets for understanding cancer biology and for therapeutic intervention in many tumors, including gastrointestinal system cancers [17,18,19], as well as lung [20,21], prostate [22,23], breast [24,25], and central nervous system cancers [26,27,28].

Ion channels unquestionably play a vital role in several characteristics of cancer, and “oncochannelopathies” for calcium, sodium, and chloride ion channels have been described [3,29]. Moreover, it is well known that potassium channels are essential for cell proliferation [30,31], and potassium channel activity was increased in early investigations of viral infection leading to oncogenic changes [32]. Furthermore, a subunit of the large-conductance voltage and calcium-activated potassium channel (KCNMA1) revealed increased expression in the course of an extensive investigation of breast cancer tissue microarrays, an observation that may be related to the cancer’s high rate of proliferation and malignancy [33].

Potassium channels exist virtually in almost all species except some parasites and perform crucial roles. They are, by far, the largest, most diverse, and well-studied family of ion channels and include four subfamilies: voltage-gated K^+^ channels (Kv), Ca^2+^- and Na^+^-activated K^+^ channels (KCa, KNa), inwardly rectifying K^+^ channels (Kir), and two-pore domain K^+^ (K2P) channels [34]. These subfamilies differ by domain structure, gating mechanisms, and functions [35]. Potassium channels have been a major focus in oncology owing to their role in cell proliferation, differentiation, regulating cell volume, and maintaining membrane potential. The expression of an exhaustive array of potassium channels varies not only by cell type but also from normal to metastatic cells [36]. Several types of Kv channels are known to play a critical role during apoptosis due to their involvement in cell-cycle progression, resting membrane potential, and volume regulation, rendering them a potential molecular target in the diagnosis and therapy of some cancers [37]. Numerous studies have identified dysregulated potassium channel expression across many tumor types, including breast, prostate, lung, endometrium, pancreas, and others, and were extensively reviewed elsewhere [35]. In summary, the increased expression of potassium channels in cancer is associated with metastasis and tumorigenesis [38,39], higher-grade tumors [40,41], severe cancer phenotypes [42,43], cancer cell migration [44,45], proliferation through calcium regulation [46,47,48], and lower overall survival [49,50], among other effects. As such, the altered potassium ion channel expression serves central roles in neoplastic transformation and provides a toolkit that diverges from the healthy counterparts and warrants thorough investigation as a prevailing focus in cancer biology and therapeutics.

## 3. The Disease Burden of Glioma

As the most prevalent primary intracranial cancer, glioma represents over 80% of all brain tumors [51]. Although relatively rare, the incidence of glioma varies significantly by histologic type, age at diagnosis, gender, race, and country [51]. Generally, the overall age-adjusted incidence rate for all gliomas is about 6.0 per 100,000 population [52] or 250,000 new diagnoses per year worldwide [53]. According to data in 2022, glioma is more prevalent in older adults, with a peak incidence between 45 and 65 years of age. Nevertheless, gliomas are one of the most common solid tumors in children, accounting for over 45% of tumors among the age group of 0–19 years [54]. Gliomas derive their name from their originating cells, glial cells that support other cells of the brain, in contrast to nonglial tumors, that instigate from other brain structures, including nerves, blood vessels, and glands [55]. Based on the type of cell where they start, gliomas are further classified into astrocytomas, developed from star-shaped astrocytes that make up the larger part of the supportive brain tissue, oligodendrogliomas, originating from oligodendrocytes that produce the myelin sheath, and ependymomas, arising within the posterior fossa and supratentorial regions of the brain, as well as in the spinal cord. The median survival remains about 2–5 years for such gliomas [56,57]. On the other hand, glioblastoma multiforme (GBM) accounts for about 60–70% of all gliomas and is the most invasive and rapidly growing type of glial tumor. It originates from the anaplastic degeneration of different cells, including astrocytes, oligodendrocytes, and neural stem cells [58]. The most frequent malignant primary tumor of the central nervous system is GBM, with typical survival of 9 to 16 months, 2-year survival below 25%, and 5-year survival of 6.8% only, despite advancements in neurosurgery, radiation therapy, and chemotherapy. GBM is, therefore, regarded as one of the most fatal tumors, with a major problem in its treatment being the high resistance to chemotherapy and irradiation [59,60]. Despite tireless efforts over the past 20 years to create new treatment modalities for GMB, achieving long-term remissions in clinical trials is still remote, leaving only a few treatment options [61]. Studies show that GBM has a slight male predominance, with a male-to-female ratio of approximately 1.4:1 [62]. The clinical presentation of GBM can vary greatly depending on the stage and location of cancer, with symptoms including slow progressive neurologic deficits, usually motor weakness, in addition to commonly reported headache, nausea and vomiting, cognitive impairment, and seizure [63]. A significant burden is placed on the healthcare system, as well as on individual patients, for the treatment of this disease. Estimates of the median expenditure for an individual undergoing glioma treatment in 2019 was over $184,000, with radiation therapy accounting for the majority of this cost [64].

According to malignancy level, primary brain tumors are usually rated on a scale of I to IV, with increasing grade corresponding to higher malignancy. Gliomas are categorized as either low-grade (I and II) or high-grade (III and IV), whereas high-grade gliomas are also termed malignant or anaplastic gliomas. The latter is known for displaying high rates of mutations such as TP53, EGFR, or PTEN, which correlate with poor prognosis. All grade IV gliomas are glioblastomas [55]. Furthermore, the 2016 World Health Organization (WHO) classification of brain tumors classifies glioblastomas based on the mutational status of isocitrate dehydrogenase 1/2 (IDH). Most glioblastomas are IDH-wildtype (wt), which typically arise in patients aged over 50 years and are associated with poor prognosis. Only about 10% of glioblastomas are IDH-mutant (mut), which are often secondary tumors that arise from the progression of lower-grade gliomas and are associated with better survival compared to IDH-wt [65].

The major hindrance with malignant glioma remains its high migratory and invasive potential into the healthy brain parenchyma, avoiding the possibility of total surgical resection of tumor cells. Despite treatment, gliomas normally recur at or near the surgical site, establishing new tumors more resistant to further treatment, and are the primary cause of mortality. Questionably, at the time of surgery, a large number of cells have already detached from the original tumor and invaded far brain areas causing glioma metastasis [66]. Other typical features of malignant gliomas are their high proliferation rates, copious mitosis, and circumvention from apoptosis, probably due to common gene mutations that result in the dysregulation of the major growth factor signaling pathways [67].

## 4. Ion Channels in Glioma and the Importance of Potassium Channels

During oncogenic transformation, it is expected that genes encoding ion channels are affected [3]. For instance, upon microarray-assisted expression profiling of ion channel genes in breast cancer [68], lung adenocarcinoma [69,70], and glioma [71], a total of 30, 37, and 18 ion channel genes, respectively, were differentially expressed compared with normal tissues. As such, ion channel dysregulation may contribute to the pathophysiological features of different cancers, and data on their involvement in carcinogenesis, have been increasing exponentially [72]. In glioma, ion channels have been identified as promising therapeutic targets that may decrease the invasiveness of brain tumor cells [73]. Of the channels investigated, the transient receptor potential (TRP) channels and low threshold-activated calcium channels have similarly important roles in brain malignancy since dysregulated calcium ion signals profoundly affect glioma cell proliferation, migration, and invasion [74,75]. Furthermore, both chloride and potassium channels have emerged as vital gateways to facilitate cell volume changes [76] and participate in the blockade of apoptosis [77]. The movement of ions across these channels causes cytoplasmic water to move across the membrane, permitting robust shape and volume changes. Volume changes are necessary for tumor migration and, if inhibited, may block this process [78]. Calcium-activated potassium channels have a major role in such activity [79,80]. Moreover, altered expression of ion channels, especially potassium channels, conferred an invasive phenotype to GBM, and their modification significantly reduced tumor cell invasion both in vivo and ex vivo, according to the findings by Turner and Colleagues [81]. Glioma cells with a blockade of potassium channel functions through the drug temozolomide, a cytotoxic imidazotetrazine that forms O6-methylguanine, which mismatches with thymine during DNA replication, are sensitized to this drug, potentiating the antitumor effects [82]. Additionally, experiments performed on different glioma cell lines proved that novel potassium channel inhibitors induced massive cell death in vitro [59].

As such, it is evident that potassium ion channels play a hallmark role in brain cancer processes, including proliferation, invasion, migration, and angiogenesis, which are key drivers of tumor progression in glioma [35]. With the assorted body of data available on the role of potassium channels, it is imperative to precisely review the existing literature and draw conclusions for future research in this area. In the following sections of this review, the different potassium ion channels will be presented, with focused insights into their role in glioma and their contribution to oncogenesis. Additionally, targeting potassium channels in tumor membranes as an adjuvant pharmacotherapeutic option in glioma will be reviewed.

## 5. Overview of Potassium Channel Topology and Function

In both excitable and nonexcitable cells, potassium-selective channels (K^+^ channels) constitute the most numerous and diverse subset of ion channels [83]. The study of potassium channels has advanced significantly in recent years, thanks to the development of X-ray crystallography and cryo-electron microscopy (Cryo-EM) techniques [84,85]. These techniques have enabled researchers to determine the three-dimensional structures of potassium channels at high resolution, which has greatly improved the understanding of their function and regulation [84,86]. The X-ray and Cryo-EM structures of these channels provide valuable insights into the mechanisms underlying ion selectivity, gating, and regulation of potassium channels, which are essential for maintaining proper cellular function and communication. It has also opened up new avenues for the development of novel therapeutics targeting these channels [87,88]. As previously mentioned, K^+^ channels are categorized into four types based on their conductance characteristics, structural criteria, and whether they work in conjunction with a stimulus. They include Kv (KCa and KNa), Kir, and K2P channels [83,89].

### 5.1. Voltage-gated K^+^ Channels (Kv)

The largest subgroup of the K^+^ channel family is the voltage-gated K^+^ channels (Kv channels). They are encoded by 40 genes in humans and are divided into 12 subfamilies that share six transmembrane domains and are gated by voltage [90,91]. They include Kv1 (KCNA), Kv2 (KCNB), Kv3 (KCNC), Kv4 (KCND), Kv7 (KCNQ, also known as KQT), Kv10, Kv11 (KCNH, also known as ether-a-go-go-related gene [EAG]), and Kv12. These subfamilies have distinct channels (e.g., Kv1.1–Kv1.8, Kv2.1–Kv2.2, Kv3.1–Kv3.4, and so on) according to their pharmacological profile and diverse biophysical properties [92]. The remaining subfamilies, which are Kv5, Kv6, Kv8, and Kv9 channels, do not function on their own; however, they coassemble with Kv2 subunits and alter their function [91].

**Figure 1:**
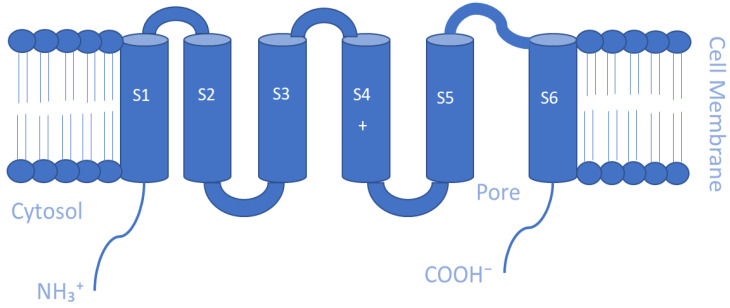
Schematic representation of the transmembrane (TM) topology of Kv channel α-subunits containing six TM segments (S1–S6), a pore region formed by S5 and S6 segments and cytosolic N- and C-termini.

Mammalian Kv channels are tetramers that are made up of α-subunits lining an ion pore. Each α-subunit possesses six α-helical transmembrane domains or segments (S1–S6), a P loop that reenters the membrane between S5 and S6, and cytosolic N- and C-termini. The S5-P-S6 segments make up the ion conduction pore known as the pore domain (PD), which is responsible for selectivity to potassium ions, and it contains a channel gate that controls ion permeation [93,94] (Figure 1). At the cytoplasmic entryway of the channel pore, the gate is composed of a collection of overcrossing α-helixes that correspond to the S6 helix of the channels [91,95]. The S1–S4 sequences that present positively charged arginine residues in the S4 helix form the voltage-sensing domains (VSDs) that are covalently linked to the pore [96,97]. These VSDs are essential for acting as voltage sensors, gating the channel and generating its opening in response to voltage changes [98,99,100]. At the membrane’s cytoplasmic side, the S4–S5 linker connects the VSDs to the PD. The VSD–PD assembly converts the membrane electric field’s potential energy into the mechanical work required for controlling potassium ions’ selective permeation [96].

In response to changes in membrane voltage, Kv channels allow the selective transport of K^+^ ions across the cell membrane, thus controlling the frequency and shape of action potentials in order to control neuronal excitability [101,102]. They perform a variety of physiological processes, such as repolarizing action potentials, setting membrane potential, determining the length or frequency of action potentials, modifying Ca^2+^ signaling and cell volume, maintaining and modulating neuronal and muscular (both cardiac and skeletal) excitability, controlling immune response and hormone secretion, and regulating cell migration and proliferation [103,104,105]. The cellular functions of Kv channels vary depending on their expression pattern and localization, and their dysfunction can lead to a wide range of pathologies, including epilepsy, cardiac arrhythmias, and neuromuscular disorders [106,107].

### 5.2. Ca^2+^- and Na^+^-Activated K^+^ Channels (KCa and KNa)

**Figure 2:**
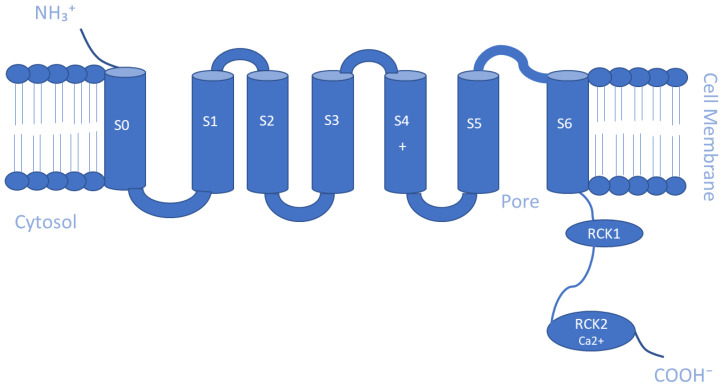
Schematic representation of the transmembrane (TM) topology of big-conductance K channel (BK) subunits containing seven TM segments with the pore region formed by S5 and S6 segments. The two regulators of K conductance (RCK) domains are in the C-terminus, with Ca^2+^ bowl in RCK2.

**Figure 3:**
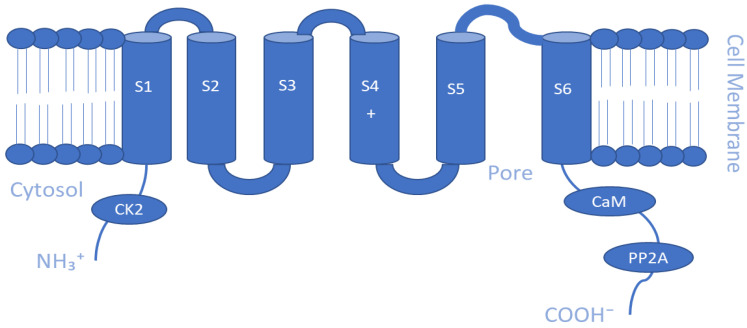
Schematic representation of the transmembrane (TM) topology of small-conductance K channel (SK) subunits containing six TM segments with the pore region formed by S5 and S6 segments. The regulators involve calmodulin and PP2A in the C-terminus and CK2 in the N-terminus.

The Ca^2+^-activated K^+^ channel family of ion channels consists of eight members that respond to Ca^2+^ concentrations and facilitate outwardly rectifying potassium currents. An increase in the intracellular concentration of Ca^2+^ shifts the voltage dependence of these channels to more negative potentials [108]. Members of this family include the following: big-conductance KCa1.1 (BK, slo1); small-conductance KCa2.1 (SK1), KCa2.2 (SK2), and KCa2.3 (SK3); intermediate-conductance KCa3.1 (IK, SK4); other subfamilies KCa4.1 (Slack, Slo2.2), KCa4.2 (Slick, Slo2.1), and KCa5.1 (Slo3) [109]. The KCa1 family is activated by both Ca^2+^ and voltage, while the intermediate conductance KCa3.1 and small conductance KCa2.1–3 channels are gated by cytosolic Ca^2+^ increase alone [110]. Furthermore, BK channels are directly activated and opened by Ca^2+^ binding without the need for calmodulin, an intermediary calcium-binding messenger protein, because they possess an extra transmembrane domain that permits the presence of two high-affinity Ca^2+^ binding sites known as a regulator of K conductance (RCK) domains [111,112], whereas, SK and IK channels are activated upon binding of calmodulin to their receptor domain in response to Ca^2+^ at low intracellular concentrations (~0.5 µM) [35]. This sensitivity to Ca^2+^ is enhanced by the additional association of the phosphorylating kinase CK2 and dephosphorylating phosphatase PP2A on the cytoplasmic face of the protein [91]. As for KCa4 and KCa5 channels, they respond instead to other intracellular ligands, such as Na^+^, Cl^−^, and pH [91]. Slo2 channels are thought to constitute the molecular basis for Na^+^-activated K^+^ (KNa) channels, which can be found in a variety of cells [113]. Similar to the Kv channels, the α-subunits of the KCa channel make homo- and heterotetrameric complexes. They have six TM segments except for KCa1 channels, where the N-terminus makes a seventh pass across the membrane ending up outside the cell [91]. Another distinctive biophysical feature of these channels is that the S4 segment of the KCa2/3 channels includes fewer charged residues than the S4 segment of the KCa1.1 or Kv channels [109]. Regarding the β-subunit of the channel, there are four types that are believed to possess regulatory functions. Beta 2 and 3 are inhibitory, while beta 1 and 4 are excitatory, and they affect the α-subunits in such a way that the channel rarely is inactivated [110]. Schematic representations of the TM topology of BK and SK channel subunits are illustrated in Figure 2 and Figure 3, respectively. These channels are expressed in various tissues and play a key role in regulating vascular and muscle tone, blood pressure, neuronal excitability, and neurotransmitter release. Therefore, they are important regulators of cellular function, and their pharmacological modulation offers potential therapeutic benefits for a range of diseases [114,115,116].

### 5.3. Inwardly Rectifying K^+^ Channels (Kir)

**Figure 4:**
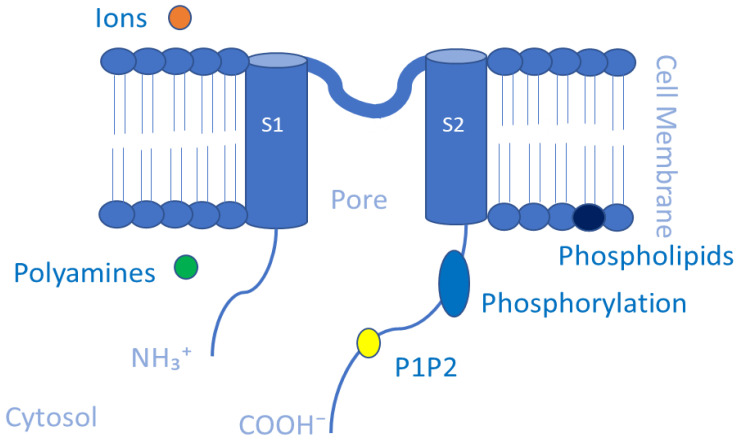
Schematic representation of the transmembrane (TM) topology of Kir channel subunits containing two TM segments with pores formed by S1 and S2. The functions of Kir channels can be regulated by small substances. The small substances are ions such as H^+^, Mg^2+^, and Na^+^ ions (in orange); polyamines (in green); phosphatidylinositol 4,5-bisphosphate (PIP2) (in yellow); phosphorylation (in blue); membrane-bound phospholipids (in black).

Kir channels are essential for maintaining K^+^ ion homeostasis and controlling cellular excitability by allowing K^+^ ions to move more easily into rather than out of the cell [117]. Numerous cell types, such as cardiomyocytes, neurons, blood cells, osteoclasts, endothelial cells, glial cells, epithelial cells, and oocytes, express Kir channels [117,118]. Such channels have diverse physiological functions based on their type and their location [118]. The mammalian Kir channel family is composed of 15 distinct genes arranged into 7 subfamilies (Kir1.x to Kir7.x) [34,92,119]. Each Kir channel consists of four homo- or heterotetrameric subunits, each of which has two transmembrane domains, cytoplasmic N- and C-termini, and an extracellular loop that serves as the pore-lining selectivity filter [117,120]. They differ structurally from Kv channels as they have only two membrane-spanning helices while lacking the four membrane helices that make up the voltage sensor in Kv channels [121] (Figure 4). As a result, various voltage-independent methods for opening and closing, including gating by G proteins, pH, and ATP, have emerged in Kir channels [118]. Kir channel subfamilies can be classified into four major functional groups: classical Kir channels (Kir2.x) that are constitutively active, G protein-gated Kir channels (Kir3.x) that are regulated by G protein-coupled receptors, adenosine triphosphate (ATP)-sensitive K(+) channels (K_ATP_ and Kir6.x) that establish the link between potassium conductance and cellular metabolism, and K(+) transport channels (Kir1.x, Kir4.x, Kir5.x, and Kir7.x) [122,123]. The functions of Kir channels can be regulated by small substances, including ions such as H^+^, Mg^2+^, and Na^+^ ions; polyamines; phosphatidylinositol 4,5-bisphosphate (PIP2); phosphorylation; membrane-bound phospholipids [124]. For example, the binding of Mg^2+^ ions to Kir channels has been shown to enhance their activity, while the binding of H^+^ ions can inhibit their activity. Polyamines such as spermine can also enhance Kir channel activity, while phosphatidylinositol 4,5-bisphosphate (PIP2) is a key regulator of Kir channel activity, with its depletion resulting in decreased channel activity [91,125]. Phosphorylation of specific amino acid residues on the channel subunits by protein kinases can also modulate Kir channel activity. Finally, membrane-bound phospholipids such as phosphatidylserine and phosphatidylethanolamine have been shown to modulate Kir channel activity by interacting with specific regions of the channel protein [126]. Overall, the regulation of Kir channels by small substances is complex and involves multiple mechanisms. Understanding the various factors that modulate Kir channel activity is important for the development of pharmacological agents that can selectively target these channels for therapeutic purposes [118,127].

### 5.4. Two-Pore Domain K^+^ Channels (K2P)

**Figure 5:**
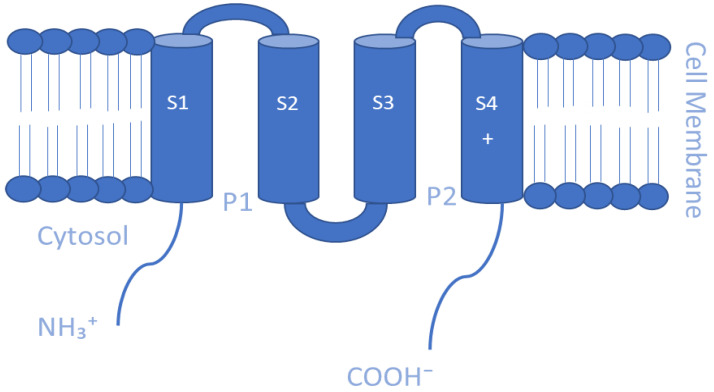
Schematic representation of the transmembrane (TM) topology of K2P channel subunits containing four TM segments with pore region 1 formed by S1 and S2, region 2 by S3 and S4 segments, and N- and C-termini located in the cytosol.

In both excitable and nonexcitable tissues, the K2P channels, a broad family of K^+^ selective ion channels, contribute to background or leak currents [128]. Mammalian K2P channels are a family of 15 members encoded by the KCNK genes that are grouped according to their functional and structural similarities into six subfamilies denoted as TREK, TALK, TASK, TWIK, THIK, and TRESK [128,129]. These channels are not voltage-gated as they lack a voltage sensor domain; however, they are regulated by several stimuli, including mechanical force, oxygen tension, polyunsaturated fatty acids (PUFAs), volatile anesthetics, acidity/pH, pharmacologic agents, heat and signaling events, such as phosphorylation and protein–protein interactions [129,130,131,132]. Therefore, as a result of their effect on the resting potential, K2P channels represent significant regulators of cellular excitability [133]. K2P channel subunits are made up of four distinct transmembrane helices (TM1-4) and two pore sequences (P), which are organized in the following order: TM1-P-TM2-TM3-P-TM4 with both the N- and C-termini located in the cytosol [133,134] (Figure 5). Hence, K2P subunits resemble a tandem assembly of two Kir channel subunits as they structurally correspond to two inward-rectifier α subunits forming dimers in the membrane, while inward-rectifier α subunits form tetramers. Another distinctive structural feature of K2P channels is the presence of the so-called “cap” structure that arises from an extended portion of the TM1 helix which serves as an extracellular ion pathway [88,135]. K2P channels play a crucial role in a variety of cellular functions, including the regulation of cardiac and neuronal excitability, the control of renal and pulmonary function, and the modulation of pain perception [136]. K2P channels are also implicated in a number of pathophysiological conditions, including hypertension, epilepsy, and chronic pain. The pharmacology of K2P channels is an area of active research, with a number of drugs and compounds being developed to target these channels [137,138,139].

## 6. Potassium Channels Dysfunction in a Tumor Microenvironment

### 6.1. Dysfunction in Proliferation and Apoptosis

The role of potassium channels is critical in carcinogenesis as it controls the processes of cellular proliferation, migration, angiogenesis, and to the contrary apoptosis, all of which determine the degree of tumor aggressiveness and pathogenesis. In this regard, alterations in the structures, mutations, sequences, and hence functions of potassium channels should be carefully addressed in tumor analysis, diagnosis, and prognosis of gliomas and other cancers.

There is mounting evidence that connects defects in K^+^ signaling and K^+^ channels to cancer, and hence blocking these channels is reported as a pharmacological mechanism to inhibit the proliferation of cancer cells [140]. Although gliomas remain very untreatable, except for surgical removal of the tumor, the abnormal expression of K^+^ channels in such tumors has triggered extensive research into potential alternative modulations to improve glioma prognosis, including the blockage of K^+^ signaling [89]. In one study by Qin Ru et al., quinidine significantly inhibited the proliferation of U87-MG cells (isolated from malignant gliomas) and induced apoptosis via the mitochondrial-dependent pathway [141]. Yet another report provided a connection between membrane depolarization, quinidine, K^+^ channel blockage, ornithine decarboxylase blockage, and inhibition of cellular proliferation in C6 glioma cells [142]. These results and others are indicative of the mechanistic role of quinidine in halting proliferation and inducing apoptosis in glioma cells by targeting voltage-gated K^+^ channels.

Interestingly, other research on K^+^ signaling in glioma cells reported that the inhibition of Kv channel opening improves the proliferative abilities of N2A cells (mouse neuroblastoma and stem cell morphology) [143]. It is thought that the role of K^+^ channels in controlling proliferation can be understood by understanding Ca^2+^ signaling and its connected role to tumorigenesis. The hyperpolarization induced by abnormal expression of K^+^ channels in glioma cells can drive an influx of Ca^2+^ into the cells and promote cell division and mitosis [144]. Moreover, several drugs related to K^+^ channels are under investigation for efficacy in gliomas and other cancers. Yang et al. demonstrated that tetraethylammonium (selective blocker of potassium channels) inhibited growth and induced cell death in two rat glioma cell lines (C6 and 9 L) [145], possibly through reactive oxygen species (ROS) generation leading to apoptosis.

A clear link between the role of potassium channels and apoptosis induction comes from the observation that the Kv1.3 was inhibited immediately after initiation of apoptosis [146], most likely due to a tyrosine phosphorylation event [147]. If anything, this indicates that shutting off potassium channels is a hallmark event in apoptosis, and hence their overexpression correlates with glioma and tumor development. In line with that is the observation that the potassium channel ether-a-go-go 1 (Eag1) is overexpressed in several cancers. To this point, Sales et al. investigated the role of Eag1 in modulating apoptosis and proliferation in glioblastoma cells. They reported that silencing Eag1 reduced the viability and proliferation of the U87MG glioblastoma cell line and increased the apoptotic rate triggered by temozolomide (TMZ) [148], clearly pointing out and confirming a role for potassium channels (Eag1) in modulation apoptosis in a glioma context. Collectively, the above observations and reports should promote further studies on targeting Eag1 to improve the prognosis and survival of glioma patients.

### 6.2. Dysfunction in Favor of Migration

When tumors proliferate with no control, the expectation is that metastasis and migration are the next levels of aggressiveness. To drive migration and metastasis, primary glioma cells need to escape the primary tumor site and associate with blood vessels and nerve paths, allowing them to intercalate into the fine spaces of brain tissues. The process of detachment from the primary tumor (in the brain) involves signaling and events that eventually lead to cell shrinkage and volume reduction. The most likely explanations include the transport of ions across the cell membranes, increasing the osmotic pressure in favor of water release from the cells, and subsequent reduction in volume and size, allowing cells to initiate the “escape” and “invasion”. The Na-K-Cl cotransporter (NKCC), as well as the Na^+^/K^+^ pump, allow a significant buildup of K^+^ and Cl^−^ intracellularly, contributing to an important electrochemical gradient and an inevitable opening of K^+^ and Cl^−^ channels to dump K^+^ and Cl^−^ outside the cells [149]. Concomitantly, water molecules are attracted to the extracellular environment leading to shrinkage. Targeting NKCC, Na^+^/K^+^ pumps, and the K^+^ channels apparently provide potential mechanisms, at least, to prevent the initiation of migration of glioma cells.

In one report, the potassium intermediate-small conductance calcium-activated channel, superfamily N, member 4 (KCNN4, also known as KCa3.1), was investigated to elucidate an important role in the ability of glioblastoma cells to infiltrate brain tissues. The overexpression of KCa3.1 in glioblastoma cells is well established and characterized in several cell lines, including the human U87MG and the murine GL261 cells [150]. Furthermore, CD133+ (stem-like) glioblastoma cells expressed higher levels of KCa3.1 [150], indicating the role of KCa3.1 in cell regeneration and the ability of stem cells to invade other tissues. On a similar note, D’Alessandro et al. xenografted GL-15 cells (human glioblastoma cells) into the brains of SCID mice, followed by a KCa3.1 blocker, TRAM-34. A 5-week follow-up revealed a significant reduction in tumor infiltration in the TRAM-34 treated mice as compared to nontreated mice [151]. Moreover, another study targeted KCa3.1 by shRNA in GL-15 cells and effectively abolished chemotaxis of the glioblastoma cells, initially shown to be driven by KCa3.1 channels [152]. Further evidence comes from the observation that KCa3.1 activity was required to attract GL-15 and U251 cells in response to SDF-1 (the stromal cell-derived factor 1, also known as C-X-C motif chemokine 12 (CXCL12)) [152]. These and other results are clearly in favor of a proinvasive role for KCa3.1 in the migration of gliomas, especially when taken together with the fact that normal human brain tissues express minimal KCa3.1 as compared to human glial tumor tissues [153]. Table 1 summarizes major findings that elucidate the type of potassium channels being manipulated in different models and types of gliomas, confirming the critical role of targeting potassium channels in future glioma therapeutics’ development.

## 7. Targeting Potassium Channels as Potential Therapeutic Adjuvant in Glioma

As mentioned previously, gliomas are the most frequent primary brain tumor in humans; however, they still have a poor prognosis when treated with the currently available therapeutic strategies [55,89]. At present, surgical resection cannot completely remove tumors given glioma cells’ invasive nature; after surgery, adjuvant radio- and chemotherapy are initiated but result in lower effective concentrations in the tumor due to the blood-brain barrier in addition that glioma cells resist proapoptotic stimuli, thus yielding a very high likelihood of residual lesions [89]. Therefore, because of all of the aforementioned reasons and in the presence of no effective treatment, new therapeutic approaches are much needed [89].

Increased evidence has shown that potassium channels are abnormally expressed in glioma cells and are key players in their growth, development, apoptosis, as well as their resistance to drugs [89,160]. As such, targeting potassium channels constitutes a potentially effective strategy for glioma treatment.

One potential approach for targeting potassium channels in glioma is through their blockage. Commonly recognized drugs such as imipramine, a well-known tricyclic antidepressant, were shown to inhibit vascular voltage-dependent K^+^ channels in a concentration- and use (closed-state)-dependent manner [161] while inhibiting PI3K/Akt/mTOR signaling and inducing autophagic cell death in glioma cells [162]. Moreover, the oral-hypoglycemic agent, tolbutamide, which is a first-generation sulfonylurea, binds to a high-affinity subunit (SUR1) of the beta-cell ATP-sensitive potassium channel resulting in the blocking of K^+^ efflux through the KIR6.2 channel [163]. Tolbutamide was shown to inhibit glioma cell proliferation by increasing connexin43, upregulating cyclin-dependent kinase (Cdk) inhibitors p21 and p27, and reducing pRb phosphorylation [164]. Furthermore, a potent short-acting insulin secretagogue, repaglinide, acts by closing ATP-sensitive potassium channels [165]. Repaglinide exhibited anticancer effects against glioma via apoptotic, autophagic, and immune checkpoint signaling [166]. In addition, the antiarrhythmic drug quinidine has been shown to block several types of K^+^ channel currents while blocking ATP-sensitive potassium channel currents in a “slow” and voltage-dependent manner [167,168]. Studies have revealed that the voltage-gated K^+^ channel blocker quinidine possessed both antiproliferative and proapoptosis effects in human glioma cells [169]. Moreover, studies have shown that the selective estrogen receptor modulator tamoxifen might have a potential chemotherapeutic effect on glioma [170]. It is proposed that tamoxifen inhibits the Kv7.2/Kv7.3 by preventing PIP2-channel interaction, but the exact mechanism of this inhibition is unknown [171]. A study demonstrated that tamoxifen has a direct action on mitochondrial complex I inhibition and, therefore, might have a chemotherapeutic effect on temozolomide-resistant glioma [172]. It also exerted cytotoxic actions and induced apoptosis in rat glioma cells in both a concentration and dose-dependent manner [173]. Additionally, clofazimine, an antimycobacterial agent, was found to block Kv1.3 channels causing massive cell death in glioma cells [59,174]. Apoptosis was induced in Kv1.3-expressing cancer cells by activating the intracellular mitochondrial pathway of this process [175]. Clofazimine was found to reduce tumor growth, proliferation, and self-renewal and acts synergistically with temozolomide to induce apoptosis [176]. These already-approved drugs present an attractive option for drug repurposing for cancer treatment since they necessitate a shorter duration than an entirely new molecule to gain approval for the new indication [35]. A summary of these drugs is presented in Table 2.

In addition, tetraethylammonium (TEA), a nonspecific potassium channel blocker, was shown to inhibit proliferation and induce apoptosis in rat glioma cell lines (C6 and 9 L) [145]. Whereas senicapoc, which has previously been in Phase III clinical trials, is made with a calcium-activated potassium channel KCa3.1 blocking tool. KCa3.1, which is widely expressed in glioblastoma, plays an important role in cellular activation, migration, and proliferation [177]. Thus, senicapoc can be available for repurposing and as a treatment option.

Moreover, novel artificial K^+^ channels have been developed by rebuilding the core modules of natural K^+^ channels in artificial systems. They can effectively be inserted into cell membranes to facilitate the transmembrane transfer of K^+^, which disturbs cellular K^+^ homeostasis and ultimately leads to cell death [178]. It is also worth mentioning that approaches based on simulation and machine learning and techniques based on static protein structures would help in investigating the dynamic nature of ion channels, including K^+^ channels, and controlling the process of rational drug design [10]. The molecular mechanisms underlying channel functioning and probable ligand-binding sites have been revealed by numerous computational methods that would aid in the introduction of a more successful drug discovery paradigm in the near future [179,180,181]. Artificial intelligence (AI) can be a powerful tool for drug discovery and development [182]. It can help identify specific potassium channels that are overexpressed in glioma cells and which can be targeted by drugs to slow or stop tumor growth. Once a target has been identified, AI can help design drugs that are more selective and potent, with fewer off-target effects [183]. It may also be used to screen large databases of approved drugs and identify those that may be effective against glioma. This approach can accelerate drug discovery and reduce the cost and time required for clinical development [182]. Therefore, the treatment of glioma by targeting potassium channels and its specific mechanism remains an area of active research, and further studies are needed to determine the effectiveness and safety of this approach.

## 8. Conclusions

In conclusion, potassium channels are known to be highly correlated with the malignancy of gliomas, and blocking these channels impacts a wide range of cellular tumor functions. Valuable information about the therapy of glioma may be gained from the effect of potassium channel inhibitors in glioma proliferation, apoptosis, and migration. However, there is currently a scarcity of experimental evidence on the function of potassium channel manipulation at the level of glioma tissue, despite the fact that the cellular mechanisms governed by potassium channels are incredibly comprehensive. An example of a future research direction in this regard may be the utilization of the potassium channels as a target for gene therapy using antisense oligonucleotides to stop tumor growth. AI as well has the potential to accelerate the development of new drugs for glioma by enabling more efficient drug discovery, personalized treatment, and drug repurposing. Therefore, more research is needed to determine how potassium channels contribute to the development of gliomas and how to translate candidate potassium channel inhibitors into the clinic.

## Figures and Tables

**Table 1:** Type of potassium channels manipulated in the associated glioma cell line/model with a summary of major related findings.

Type of Channel	Model/Cells	Related Major Findings	**References**
EAG1	Glioblastoma (GBM)	Overexpression of miR-296-3p sensitized glioblastoma cells to anticancer drugs by reducing EAG1 expression	[154]
hERG	Glioblastoma (GBM)	miR-133b and miR-34a were markedly downregulated in clinical GBM specimens The hERG gene was a direct target of miR-133b and miR-34a	[155]
BK	a. Glioblastoma Stem-like Cells b. Human Glioma Cell line c. U251 glioma cells	BK channels are highly expressed in GBM stem-like cells and contribute to their high migratory activity Potassium channel opener CGS7184 increased cell respiration and induced mitochondrial membrane depolarization and cell death Intracranial implantation into SCID mice, ablation of KCa3.1 with inducible shRNA resulted in a significant reduction in tumor invasion into surrounding brain in vivo	[156,157,158]
IK	Human GBM Cells	TRAM-34 (IK blocker) radiosensitized ectopic glioblastoma associated with shorter in vivo and high IK mRNA abundance patient OS in low-grade glioma and glioblastoma.	[159]
KCa3.1	a. Mouse Glioma Model b. GL261 cells	Blockade of KCa3.1 with TRAM-34 has coadjuvant effects with TMZ, reducing GL261 glioma cell migration, invasion, and colony-forming activity and increasing apoptosis KCa3.1 silencing potentiates the inhibitory effect of TMZ on glioma cell viability TMZ/TRAM-34 cotreatment increases the number of apoptotic tumor cells and the mean survival time in a syngeneic mouse glioma model	[82]
Kir6.2	Human Glioma Cells	The expression of KATP channels in glioma tissues was higher than that in normal tissues. Treatment of glioma cells with tolbutamide, a KATP channel inhibitor, suppressed the proliferation of glioma cells	[159]

**Table 2:** Approved drugs that are candidates for drug repurposing for glioma treatment.

Drug	Category	Mechanism	Effect on Glioma	References
Imipramine	Tricyclic antidepressant	- Inhibits vascular voltage-dependent K^+^ channels - Inhibits PI3K/Akt/mTOR signaling	Induces autophagic cell death	[161,162]
Tolbutamide	Sulfonylurea (oral-hypoglycemic agent)	- Binds to the beta-cell ATP-sensitive potassium channel resulting in blocking of K^+^ efflux through the KIR6.2 channel - Increases connexin43, upregulates cyclin-dependent kinase (Cdk) inhibitors p21 and p27, and reduces pRb phosphorylation	Inhibits cell proliferation	[163,164]
Repaglinide	Short-acting insulin secretagogue	- Closes ATP-sensitive potassium channels	Exhibits anticancer effects via apoptotic, autophagic, and immune checkpoint signaling	[165,166]
Quinidine	Antiarrhythmic drug	- Blocks voltage-gated K^+^ channels	Exhibits antiproliferative and proapoptosis effect	[141,142]
Tamoxifen	Selective estrogen receptor modulator	- Inhibits the Kv7.2/Kv7.3 through preventing PIP2-channel interaction - Exact mechanism unknown	Exerts cytotoxic actions, induces apoptosis, and has direct action on mitochondrial complex I inhibition	[170,171,172,173]
Clofazimine	Antimycobacterial agent	- Blocks Kv1.3 channels	Reduces tumor growth, proliferation, and self-renewal	[59,174,175,176]

## Data Availability

Not applicable.
